# Disseminated histoplasmosis diagnosed through blood smear

**DOI:** 10.1590/0037-8682-0378-2022

**Published:** 2022-12-16

**Authors:** Claudio José dos Santos, Thiago José Matos Rocha, Aryanna Kelly Pinheiro Souza

**Affiliations:** 1 Universidade de São Paulo, Faculdade de Saúde Pública, Programa de Pós-graduação em Epidemiologia, São Paulo, SP, Brasil.; 2 Universidade Estadual de Ciências da Saúde de Alagoas, Centro de Patologia e Medicina Laboratorial, Maceió, AL, Brasil.; 3 Universidade Estadual de Ciências da Saúde de Alagoas, Centro de Ciências da Saúde, Núcleo de Ciências Biológicas, Maceió, AL, Brasil.

A 28-year-old male farmer from the rural zone of Alagoas, seropositive for HIV and not adhering to antiretroviral therapy, was admitted to an infectious disease service in northeast Brazil with continuous fever, asthenia, cough with bloody expectoration, and loss of 20 kg in 30 days.

**FIGURE 1:**
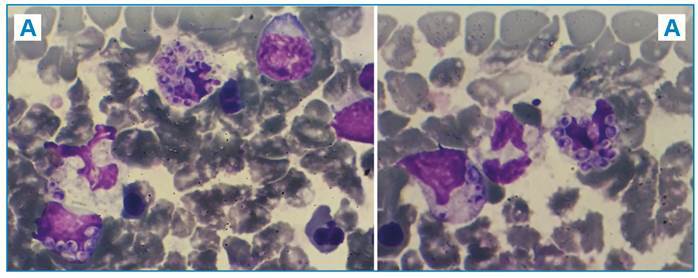
**A-B:** Numerous intracytoplasmic yeast cells observed within neutrophils (arrow) in peripheral blood with morphology suggestive of *Histoplasma capsulatum*. 100X immersion oil observation.

On physical examination, he had hepatosplenomegaly, jaundice 3+/4+, dyspnea, SaPO_2_ 93%, and whitish plaques on the tongue. Complementary tests revealed upper gastrointestinal bleeding on endoscopy; interstitial infiltrate, lymph node enlargement, and bronchial thickening on chest radiography; and visceromegaly on abdominal ultrasound. The laboratory profile revealed severe anemia and thrombocytopenia and mild leukocytosis (hemoglobin 4.8 g/dL, hematocrit 30.1%, leukocytes 11,800/mm³, platelets 45,000/mm³), besides intracellular structures in neutrophils suggestive of *Histoplasma capsulatum* in the blood smear (Figure 1A and 1B ). The biochemistry was suggestive of an active inflammatory process (CRP 1040.0 mg/dL, DHL 1166 U/L), CD4 23/mm³. Broad-spectrum antimicrobial chemoprophylaxis for opportunistic pathogens was initiated; however, the patient progressed to a serious general condition and died from acute respiratory failure and septic shock in the second week of hospitalization.

Histoplasmosis develops in immunodeficient individuals, such as those affected by acquired immunodeficiency syndrome (AIDS), with a high rate of morbidity and mortality^1^ ^,^ ^2^. The disseminated subtype addressed in this work is a strain with poor prognosis, and its diagnosis is still challenging to determine^3^. 

As definitive diagnosis of histoplasmosis can be difficult and time-consuming, the need to expand access to other tests becomes relevant and may be useful in deciding the initiation of empirical therapy for histoplasmosis. Thus, in the present case description, we highlight the relevance of performing microscopic examination of peripheral blood smear, allowing direct viewing of the fungus and the final diagnosis of histoplasmosis.
